# Exploring Features of the Pervasive Game Pokémon GO That Enable Behavior Change: Qualitative Study

**DOI:** 10.2196/15967

**Published:** 2020-05-25

**Authors:** Jonne Arjoranta, Tuomas Kari, Markus Salo

**Affiliations:** 1 University of Jyväskylä Jyväskylä Finland; 2 Institute for Advanced Management Systems Research Turku Finland

**Keywords:** Pokémon GO, digital gaming, behavior change, pervasive games, augmented reality games, location-based games, exergames, behavior change support system

## Abstract

**Background:**

Digital gaming is one of the most popular forms of entertainment in the world. While prior literature concluded that digital games can enable changes in players’ behaviors, there is limited knowledge about different types of behavior changes and the game features driving them. Understanding behavior changes and the game features behind them is important because digital games can motivate players to change their behavior for the better (or worse).

**Objective:**

This study investigates the types of behavior changes and their underlying game features within the context of the popular pervasive game Pokémon GO.

**Methods:**

We collected data from 262 respondents with a critical incident technique (CIT) questionnaire. We analyzed the responses with applied thematic analysis with ATLAS.ti (ATLAS.ti Scientific Software Development GmbH) software.

**Results:**

We discovered 8 types of behavior changes and 13 game features relevant to those behavior changes. The behavior changes included added activity in life, enhancing routines, exploration, increased physical activity, strengthening social bonds, lowering social barriers, increased positive emotional expression and self-treatment. The game features included reaching a higher level, catching new Pokémon, evolving new Pokémon, visiting PokéStops, exploring PokéStops, hatching eggs, fighting in gyms, collaborative fighting, exploiting special events, finding specific Pokémon, using items, Pokémon theme, and game location tied to physical location. The behavior changes were connected to specific game features, with game location tied to physical location and catching new Pokémon being the most common and connected to all behavior changes.

**Conclusions:**

Our findings indicate that the surveyed players changed their behaviors while or after playing Pokémon GO. The respondents reported being more social, expressed more positive emotions, found more meaningfulness in their routines, and had increased motivation to explore their surroundings.

## Introduction

### Background

Digital gaming has become one of the most popular forms of entertainment in the world [1]. As individuals engage with digital games, they are receptive to potential changes in their behavior [2]. This is the case especially of pervasive games, which refers to games played in physical locations [3].

The characteristics and popularity of digital games make them a strong potential platform for behavior change, or the modification of human activities and routines. More specifically, digital games can offer new ways to motivate individuals to change their behavior for the better and play a part in combating contemporary problems such as a sedentary lifestyle and social isolation [4,5]. This is in line with the recent notion of gamification, which refers to implementing game elements to human activities [6] with the aim of motivating people to behave in favorable ways through more gameful and enjoyable user experiences [7].

Literature on digital games and other potential behavior change support systems (BCSSs) has provided valuable first insights on the link between games and behavior change [8-10]. However, we could not locate any studies that (1) explore the various behavior change types digital games enable or (2) identify the game features driving different types of behavior change. We aim to address this research gap by focusing on the context of pervasive games because of their potential for inducing a wide range of behavior changes. Our research question is: What kinds of pervasive game features drive different types of behavior change?

This study uses a qualitative research approach given its usefulness in tapping into individuals’ real-life behaviors and exploring new areas of investigation [11,12]. We desire to extend the prior literature by exploring pervasive games as BCSSs and uncovering insights about specific game features and their relation to behavior change types. As for practical implications, our findings can help game designers and public sector parties identify game features that influence people’s behavior for the better.

### Theoretical Background

#### Pokémon GO

Pokémon GO [13] was launched in July 2016 and gained tremendous success around the globe [14,15]. As is typical for mobile games, after its success peaked, the number of downloads and players of Pokémon GO declined [16,17]. Nevertheless, in early 2019 the game still had a large player community and made exceptional profits, with an estimated revenue of $795 million worldwide in 2018 alone [18]. There are several aspects that drive Pokémon GO’s popularity and success compared with earlier location-based and pervasive games (eg, franchise and fandom [19], game enjoyment, ease of use, challenge, nostalgia [20], a critical mass of players, social play, game mechanics, and the combination of outdoor play and physical activity [21]). The findings from Kari et al [22] suggest that perceived positive behavior changes are also one reason behind playing Pokémon GO.

The game itself is a location-based mobile game based on augmented reality mechanics. Since its publication, Pokémon GO has undergone several updates and received new content and features [23]. Yet the basic idea remains the same: The player navigates physical world settings, and the player’s avatar follows on an in-game map based on real street maps. The in-game map includes some central places concerning gameplay, such as PokéStops (locations where players can collect game items) and gyms (locations where the players can battle others with their Pokémon). When the game is played as intended, it requires moving around physical world places, thus requiring physical activity [24]; as such, the game has an exergaming character [25]. The physical world locations of Pokémon GO are widely based on Niantic’s earlier location-based game Ingress [13,26,27].

Academic interest in different aspects of Pokémon GO is extensive [28,29]. Several studies concerning Pokémon GO have reported on the game’s positive influence on physical activity behavior [29-35]. However, other studies have questioned the sustainability of this increased physical activity [29,33]. Yet other studies have concluded that Pokémon GO can support people in changing their sedentary screen time and indoor activities to outdoor activities [35-37]. Pokémon GO also seems to have a strong social element. Research suggests the game can change people’s social behavior [29] and aid with social withdrawal issues [38,39]. However, beyond these many reported positive side effects and benefits, there have also been reports of hazards connected to playing Pokémon GO [29,32,40], such as accidents, injuries [41], trespassing [42], and potential geographically linked safety risks [43]. Hence, the game could also change behavior in negative ways, which, of course, is important to prevent.

There are several motivations to play Pokémon GO (eg, exercise, fun, escapism, nostalgia, friendship maintenance, relationship initiation, and achievement [44]). The chief motivation to play seems to be fun [34,45,46], although combined fun and exercise is also popular [45].

All in all, a scoping review by Baranowski and Lyons [29] shows that Pokémon GO–related research has focused on numerous different aspects of the game. Still, there are limited studies on multifaceted behavior change types and game features driving them within Pokémon GO and other pervasive games. Additional research on how games can be designed to change behavior is thus necessary [29].

#### Behavior Change Support Systems

Digital games and other information technology (IT) can influence people’s attitudes and behaviors [47]. This impact can be intended or unintended (eg, a side effect due to design). IT applications can be developed to induce behavioral changes both positive (eg, supporting a desired behavior change) and negative (eg, deception or coercion) [47]. The concept of BCSSs is defined as a “sociotechnical information system with psychological and behavioral outcomes designed to form, alter, or reinforce attitudes, behaviors, or an act of complying without using coercion or deception” [47]. Hence, BCSSs emphasize the voluntariness of behavior change. BCSSs can function in 3 ways [47]: formation, alteration, and reinforcement outcomes. The formation outcome means “formulation of a pattern for a situation where one did not exist beforehand,” alteration outcome involves “changes in a person’s response to an issue,” and reinforcement outcome refers to “the reinforcement of current attitudes or behaviors, making them more resistant to change.”

Further, Oinas-Kukkonen [47] divided changes into 3 categories: attitudes, behaviors, and the act of complying. The goal with systems supporting changes to attitude is to “influence the end users’ attitudes rather than behavior only,” with systems supporting changes in behavior, it is to “elicit a more enduring change than simple compliance once or a few times,” and with systems supporting change in an act of complying, it is “simply to make sure that the end user complies with the request of the system” [47].

With BCSSs, there are 3 types of stakeholders with the intention of influencing someone’s attitudes or behaviors: those who create or produce the BCSS, those who distribute or grant access to the systems, and the individual users of the systems [47,48]. The focus of this study is on the users, but it also conveys implications for the other 2 stakeholders.

There are various ways that digital games can act as BCSSs. For example, by playing digital games, players are exposed to different kinds of game experiences that can function as stimulants for the players’ future behaviors [49]. According to Oinas-Kukkonen [47], ideal BCSSs persuade users to adopt intended behaviors voluntarily; however, changes in behavior can also be unintentional. For further reading about behavior change in general and related techniques, we refer the reader to research conducted by Connolly et al [49], Michie et al [50,51], and Davis et al [52].

#### Pervasive Games

While most digital games are played inside and on stationary devices, there is a tradition of games that are not tied to one location. These are referred to as pervasive games [53] and include diverse types of games from alternative reality games to those played on mobile devices. Games are identified as pervasive when they expand the spatial, social, or temporal limits of play beyond what is typical to them [3].

For example, I Love Bees [54] was an alternative reality game created as part of a viral marketing campaign for Halo 2 [55]. I Love Bees required players to collaborate in a wide geographical area. Another game that used mobile technology is BotFighters [56], in which players fought each other’s robots by sending text messages. Further, games like Momentum [57] removed players from their ordinary lives and gave them a fictional framework in which to participate.

Pervasive games blur different contexts and mindsets, requiring a variety of activities and approaches [53]. However, location-awareness technology and casual games have paved the way for less intensive approaches to pervasiveness. Presumably these also affect player behavior, but previous research has not explored possible behavior changes in depth. We investigate one such example, Pokémon GO.

## Methods

### Data Collection

We employed a qualitative approach in our study due to its benefits in exploring new areas and examining users’ real-life behaviors [12,58]. More specifically, we used the critical incident technique (CIT) [59], which focuses on acquiring individuals’ descriptions of their actual behavior. CIT has been widely used in studying individuals’ experiences and behaviors related to products and services [60], with the term critical incident referring to a single experience that a person perceives as unusually positive or negative [61]. CIT has several advantages: it focuses only on meaningful incidents that are typically influential for human behavior; respondents use their own words instead of research terminology; critical incidents are easy to remember and describe; and the incident descriptions tend to reflect detailed accounts of activities and behaviors [59,60,62,63]. In summary, CIT is considered an established technique for collecting data and exploring human behavior [60,64].

We collected data by conducting an online questionnaire in English for an international audience. We collected the data right after Pokémon GO was released, from mid-July to mid-November 2016. In seeking a broad range of respondents, we distributed the questionnaire via different kinds of online forums (eg, related to gaming, Pokémon GO, lifestyle, wellness, culture, cooking, sports, cuisine) and social networking services (Facebook, Twitter, and Reddit). There were no rewards offered for responding to the questionnaire. To elicit descriptions of outstanding incidents, we adapted wordings from recognized CIT studies [64,65] such as, “Think of a time when you had an outstandingly positive or negative experience [with Pokémon GO].” To acquire further details about the given incident, we asked a series of open-ended questions, including “Explain what you were doing and what happened.” “What exactly caused the positivity/negativity of the experience?” “Why do you consider this to be an outstanding experience for you?” and “How did you perceive the game and/or the gaming situation before and after the experience?” Each respondent of the CIT questions was required to have experience playing Pokémon GO. This was ensured by asking the respondents, “Have you played Pokémon GO?” Additionally, the questionnaire included questions for another study. The full question items are available from the authors by request. More details on the questionnaire can be found in the Checklist for Reporting Results of Internet E-Surveys (CHERRIES) [66] in Multimedia Appendix 1.

### Data Analysis

We used applied thematic analysis [67] and the software ATLAS.ti (ATLAS.ti Scientific Software Development GmbH) to qualitatively analyze the data. During the analysis, we took suggestions from Charmaz [68] into account. To ensure the quality of the empirical evidence, we followed guidelines suggested by Gremler [60] and Bitner et al [65] and set these criteria for the reported incidents to be included in the analysis: it was required to be a single incident concerning a specific playing session of Pokémon GO with sufficient incident description. Altogether, we received 270 complete responses, of which 262 met the inclusion criteria.

In the first phase, the 262 CIT responses were free-coded by one of the authors. All responses were read through twice, after which a set of preliminary codes was formed. We tested these codes on the first 20 responses and on the basis of these responses revised the codes. We then coded all of the responses, with new codes introduced when responses did not fit the original categories. Finally, we merged some codes upon noticing more general trends or that the codes did not include sufficient enough responses to be informative. After the initial coding, results were cross-checked by another author. This resulted in small refinements in a few category names and 19 detailed coding suggestions that were decided on via consensus among the authors. Altogether, we had 70 codes reflecting 1314 quotations (for a full list of codes, see Multimedia Appendix 2). We then categorized the codes into more general categories, resulting in 8 categories of behavior change types. Because each quote could be coded with multiple codes, they could belong to multiple categories. The quotes are presented in this study with slight corrections to style and grammar.

In the second phase, we identified game features from Pokémon GO. We wished to understand how game features relate to different types of behavior change, so we analyzed the links between game features and behavior change.

Our analysis was built upon game design elements found by Meschtscherjakov et al [34] when they “analyzed the Pokémon GO app by systematically evaluating each possibility to interact with the app.” They focused on elements they deemed as persuading “players to be more physical [sic] active.” They “do not claim that this list is comprehensive, since the different game design elements overlap each other, and Pokémon GO is constantly updated with new features.” The elements identified by Meschtscherjakov et al [34] were as follows:

- Reaching a higher level
- Catching new Pokémon
- Evolving new Pokémon
- Visiting PokéStops
- Exploring PokéStops
- Hatching eggs
- Fighting in gyms
- Collaborative fighting
- Exploiting special events

Because we were interested in behavior changes besides physical activity, we tried to identify game features missing from the list. By analyzing the answers in our data, we found 4 additional features:

- Finding specific Pokémon: Not all Pokémon are the same, as some are rarer or more powerful than others. Players care about which Pokémon they find.
- Using items: Players use different items during gameplay that either make playing the game easier or enable new actions. Lures are one of the most important items that change the social dynamic of the game.
- Pokémon theme: Pokémon GO is Pokémon-themed, but that is not a necessary quality. It could also have some other theme, and players could react differently to it.
- Game location tied to physical location: Pokémon GO is a location-based pervasive game. There are other Pokémon games that are not tied to specific locations, and players interact differently with these games.

After we identified these game features, we compared them to the behavior change categories found in the previous phase. We considered there to be a link between a game feature and a behavior change if either of 2 conditions was met: (1) the link was explicitly mentioned in the data (“I walked around the block to hatch my eggs”) or (2) the link was likely based on our knowledge of the game and the behavior described in the data (“I continued to play because I wanted to get a Pidgeot.” Pidgeots are Pokémon evolved from Pidgeys, suggesting a link to the game feature evolving new Pokémon).

We identified the most central game features (those that clearly stood out above the others based on frequency) that drove players toward each specific behavior change by counting the coded links between game features and behavior change. The exact quantity of the central links varied between different categories of behavior change, ranging from 3 to 5. These included 2 game features that appeared important for all behavior change categories.

## Results

### Summary

Our results comprise responses from 262 Pokémon GO players. Table 1 summarizes the background information of the respondents. A total of 78.2% (205/262) of the reported incidents were positive and 21.8% (57/262) negative. The incidents reflect 8 types of behavior changes, which we present in the following subchapters with the related game features that drive such changes.

On a general level, there were 2 game features behind all behavior changes: game location tied to physical location and catching new Pokémon. Thus, these are the 2 most central features that supported the respondents’ (intentional or unintentional) behavior changes. The reason why these 2 features are so influential regarding behavior changes is their strong connection to Pokémon GO’s fundamental game mechanics (merging the game world with the physical world) and purpose (catching new Pokémon). The following quotes illustrate how these features drove behavior changes.

It [Pokémon GO] is an excellent motivator for my husband and I to exercise and walk at local parks. We bring our daughter and have loads of fun.

Respondent 57; game location tied to physical location

I walked around catching Pokémon and visiting PokéStops and walked like 10k in 2 days. I usually struggle to even leave the house.

Respondent 22; catching new Pokémon

Certain game features were not important for any type of behavior change. They were reaching a higher level, evolving new Pokémon, collaborative fighting, and exploiting special events [34]. This does not necessarily mean that these are not important features of the game, just that they were not associated with changes in the respondents’ behavior.

**Table 1:** Sociodemographic characteristics of the sample (n=262).

Characteristics		Value, n (%)
**Gender**		
	Male	82 (31.3)
	Female	176 (67.2)
	Other	4 (1.5)
**Age in years**		
	<20	26 (9.9)
	20-29	151 (57.7)
	30-39	47 (17.9)
	≥40	38 (14.5)
**Employment status**		
	Student	105 (40.1)
	Employee	120 (45.8)
	Entrepreneur	10 (3.8)
	Pensioner	10 (3.8)
	Other	17 (6.5)
**Highest education**		
	Comprehensive school/primary education	12 (4.6)
	Vocational school (applied)	11 (4.2)
	Upper secondary school/gymnasium	55 (21.0)
	Tertiary education at university of applied science	36 (13.7)
	Bachelor or master’s level at university	128 (48.9)
	Licentiate or doctoral level	11 (4.2)
	Other	9 (3.4)

### Added Activity in Life

One way Pokémon GO affected the respondents’ routines was giving them more things to do. The respondents reported that while many of their existing activities were centered around their homes, Pokémon GO gave them a reason to leave their homes. Importantly, the respondents played the game for short bursts whenever it was convenient. They had no need to plan ahead how to incorporate Pokémon GO into their schedules and instead could play whenever a suitable opportunity presented itself.

The game features that drove respondents toward added activity in life were visiting PokéStops and Pokémon theme. Visiting PokéStops enabled added activity, since the PokéStops offered new potential locations for visitation. For instance, a cluster of PokéStops persuaded one player to walk around a waterfront during sunrise instead of going home after a night shift. Familiarity with the Pokémon theme was another trigger for respondents to deviate from their previous activities:

Pokémon was a big part of my childhood and the familiar characters that I loved as a kid helped me to motivate myself enough to go out and move around.

Respondent 239

### Enhancing Routines

Several respondents reported how Pokémon GO became part of their usual routines. They continued going through their daily routines (eg, walking the dog, going to work, buying groceries), but these routines were enhanced by the addition of activities related to Pokémon GO. The routines themselves did not change, but they became more enjoyable. Being able to play Pokémon GO in short sessions while doing something else was key to this type of game engagement.

The game features that drove the respondents toward enhancing their routines included hatching eggs and finding specific Pokémon. The respondents expressed that they easily complemented their walking routines with the goal of hatching eggs. For example, one of the respondents stated, “I love walking if I have a goal, I cannot stand walking without a goal.” Similarly, the respondents complemented activities such as walking home from work by looking for new or otherwise meaningful Pokémon. They described how finding such Pokémon brought an additional layer of enjoyment to otherwise boring routines.

### Exploration

Pokémon GO encouraged the respondents to explore their surroundings. For instance, the respondents reported that they intentionally set out to find specific PokéStops. Some even reported that they chose to use new paths to familiar places because of the game, making them more aware of their usual surroundings. In addition, a few respondents described how the game led them to explore their surroundings unintentionally while looking for a Pokémon.

Exploration was linked with the game feature exploring PokéStops. The respondents expressed that going around different PokéStops was a good way to find new and interesting places both in their hometown and when traveling. As PokéStops are typically located at landmarks, the respondents became curious to see landmarks in the physical world. Thus, because PokéStops are located at these particular spots, they provided value besides just collecting items.

Seeing completely new things in my surroundings, all because of the game had PokéStops singling them out.

Respondent 76

We were on holiday, visiting another city, and walked around seeing places and playing.

Respondent 206

### Increased Physical Activity

One of the central changes in behavior reported was an increase in physical activity. Most respondents played Pokémon GO for both fun and as a form of exercise. While some claimed that the game was a good motivator to exercise more, others said the increase in physical activity was an unintentional but welcome side effect of playing Pokémon GO.

Two game features that drove the respondents toward increased physical activity were hatching eggs and Pokémon theme. The walking distance required to hatch an egg led the respondents to walk or run more (eg, “I’m walking 5k a day at least, [for] them eggs”). The game’s theme (ie, being about Pokémon) also increased physical activity: the respondents reported feelings of interest, fandom belonging, and nostalgia toward Pokémon as a trigger to be physically active or make exercising more fun with the game.

Being a huge fan of Nintendo and Pokémon [...] I was extremely excited to try it out considering how it might get me more motivated to exercise and have fun at the same time!

Respondent 27

I love that I finally get to go on adventures outside, get some fresh air and exercise, AND play a game from a series I’m fond of (and have grown up with my entire life).

Respondent 63

### Strengthening Social Bonds

One-third of the respondents played Pokémon GO alone, meaning that most played it with others: friends, children, parents, or significant others. Based on the responses, Pokémon GO made it easy for the respondents to spend quality time with people important to them. The respondents even described how playing the game was made more meaningful when the experience was shared with others. However, the highly social nature of playing Pokémon GO made a few respondents potentially risk becoming outsiders, as they reported playing only because it was expected of them.

The game features behind strengthening social bonds included finding specific Pokémon, Pokémon theme, and using items. The moment of capturing a rare Pokémon not only delighted the respondents but made some of them happy for each other (eg, “Catching rare [Pokémon] and being happy for friend too”). Many of the respondents described their shared interest in the Pokémon theme, which increased their feelings of affinity. Last, some of the respondents perceived using items (eg, lures) as social favors and acts of goodwill, thus forming positive impressions of other players. However, using items also triggered negative feelings about free-riders (people not contributing to but taking advantage of others’ lures).

### Lowering Social Barriers

The respondents reported that Pokémon GO made socializing with strangers possible, giving the players something they could share with people they did not know. This extended to people who were often in close physical proximity, like neighbors. Another thing the respondents remarked on was that Pokémon GO erased or lowered social barriers between people of different ages: the respondents themselves ranged from students to pensioners, and they described how the game made them socialize with players of all ages. Pokémon GO supports this by dividing players into teams, focusing play around specific locations, and having items that also help other players. A small minority of respondents encountered problems with the lowered social barriers, however, reporting how strangers made them feel uncomfortable.

Two game features that lowered social barriers were fighting in gyms and using items. The items that appeared to all players (eg, lures) brought respondents together momentarily and offered an easy way for them to interact with other players. Similarly, the respondents reported that they teamed up with other players in gyms, making them natural spots for starting a conversation with unknown players. For instance, one player described, “I am forty, so I was quite happy to meet a coplayer even ol[der] than I am. We met at a gym.”

### Increased Positive Emotional Expression

Pokémon GO play often had positive emotional connotations for the respondents. They reported how they expressed positive emotions, like happiness or positivity, while playing. They also described activities that have a positive emotional valence, like laughing, smiling, and being silly. These emotions were not reserved to interactions between close friends, and the respondents described how happiness or joy spread among strangers playing together as well. Sometimes this was associated with people teaching each other how to play the game and discussing how different features of the game work.

More specifically, positive emotional expression linked to the game feature finding specific Pokémon. According to the data, encountering a favorite, rare, or otherwise meaningful Pokémon triggered emotional reactions of excitement. In many cases, this was because the appearance of a specific Pokémon was a surprise that the respondents could not foresee.

The excitement, the reaction of my friends getting excited, and high fiving [sic] when we caught a great Pokémon :).

Respondent 103

### Self-Treatment

Some of the respondents also wrote of intentionally using Pokémon GO to treat some aspect of their health, ranging from weight loss and improving fitness or mood to finding ways to use the game for help with anxiety or depression. Not all of the respondents were equally serious about this use of Pokémon GO for self-treatment, but at least for some respondents it was a part of their overall health management.

In addition to game location tied to physical location and catching new Pokémon, which were found to be behind all behavior changes, a game feature that aided self-treatment was hatching eggs. It was connected to reaching goals related to physical activity.

I combine it with my FitBit in order to maintain mobility (I have 2 types of rheumatism and fibromyalgia). I get my steps on the FitBit by hunting Pokémon.

Respondent 64

The specific game features that drove the identified behavior changes are summarized in Table 2. Many of the specific features are linked with several different types of behavior change.

**Table 2:** Features of Pokémon GO and their related behavior change types.

Feature	Behavior change type							
	Added activity in life	Enhancing routines	Exploration	Increased physical activity	Strengthening social bonds	Lowering social barriers	Increased positive emotional expression	Self-treatment
Catching new Pokémon	✓	✓	✓	✓	✓	✓	✓	✓
Visiting Pokéstops	✓							
Exploring Pokéstops			✓					
Hatching eggs		✓		✓				✓
Fighting in gyms						✓		
Finding specific Pokémon		✓			✓		✓	
Using items					✓	✓		
Pokémon theme	✓			✓	✓			
Game location tied to physical location	✓	✓	✓	✓	✓	✓	✓	✓

## Discussion

### Principal Findings

To extend previous studies that have presented initial insights about games and behavior change [9,10], this study set to explore the various types of behavior changes and the game features that drive these changes. We found 13 game features that drove behavior change in 8 different categories, demonstrating that pervasive games can lead to various types of behavior changes. In line with BCSS literature [47,69], the behavior changes that arise from pervasive games can be either intentional or unintentional. Further, a pervasive game such as Pokémon GO can influence behavioral outcomes in all 3 ways: formation, alteration, and reinforcement of behaviors. Our findings are in line with previous findings on the physical [30-35], psychological [32], and social aspects [38] of Pokémon GO. However, while these previous studies proposed potential behavior changes that Pokémon GO could induce, our study provides empirical evidence that demonstrates how the game has enabled players to change their behavior in various ways. Moreover, our study identified which game features drive these changes.

Our 8 categories of behavior change can be abstracted further: they relate to either social behavior, people’s routines, or their drive for self-improvement (see Table 3).

**Table 3:** Behavior change categories.

Pokémon GO behavior changes	Behavior change category	Game features
Strengthening social bonds, lowering social barriers, increased positive emotional expression	Social	Catching new Pokémon, fighting in gyms, finding specific Pokémon, using items, Pokémon theme, game location tied to physical location
Added activity in life, enhancing routines, exploration	Routines	Catching new Pokémon, visiting Pokéstops, exploring Pokéstops, hatching eggs, finding specific Pokémon, Pokémon theme, game location tied to physical location
Increased physical activity, self-treatment	Self-improvement	Catching new Pokémon, hatching eggs, Pokémon theme, game location tied to physical location

Most of the game features in Pokémon GO that linked to behavior change are related to the game being about Pokémon. This could be interpreted as the game designers’ success in designing the game so its central activities are well integrated in its theme. This is also apparent in our questionnaire data, with many respondents discussing things related to Pokémon being a wide-ranging transmedia franchise. The respondents grew up watching Pokémon being caught, hatching, and fighting against each other, so they expect these activities from a game about Pokémon. One lesson from our research is that pervasive games’ themes should be reflected in their mechanics, which seems to be a new finding in BCSS research. Indeed, a recent review identified 93 behavior change techniques, but none were related to theme [70].

In our study, in addition to game location tied to physical location, the two game features not specifically related to Pokémon (using items and collaborative fighting) both relate to collaboration. It is possible that Pokémon GO’s theme drew in players more interested in collaboration than competition or that once players started playing, their behavior was affected. Our respondents noted how the game created possibilities for positive social encounters. In media discourse, digital games are often considered conducive to antisocial behavior [71]. In contrast, Pokémon GO strongly encourages social interaction. Another study noted that 70% of their respondents never played alone, which is in line with our findings [43]. Even when playing does not require social interaction, it can act as a social catalyst [72]. Our findings demonstrate this happens by strengthening bonds between family and friends and by creating social encounters that facilitate community-building between strangers.

One reason why playing Pokémon GO is such a social experience is because the game encourages players to openly express enthusiasm and positive emotions, which spread to other players. The respondents reported greater positive expressions when people gathered at shared locations to engage in positive activities. Previous research has also noted Pokémon GO’s positive effect on psychological well-being [32]. In our questionnaire answers, laughter and smiles were shared due to the game’s ability to support friendly collaboration (eg, sharing a lure) without the need for rivalry. This indicates that game developers can benefit from designing activities that can be shared between players without making them challenge each other.

One way that Pokémon GO encouraged social interaction was perhaps not intended by the designers, however. When the game was first released, the servers would randomly disconnect, and the game would crash or hang. There were also few tutorials for the game. Many details of its working were not obvious to new players, and the only source of information was other players: some of our respondents even mentioned asking for advice and being taught how to play by other players. These encounters did not happen with all players but considering how rare it is for strangers to teach each other new skills, this is perhaps not surprising. According to Edwards et al [70], 75% of smartphone apps for health promotion use some form of social support, but teaching other players does not seem to be a common technique.

Pokémon GO changed respondents’ daily routines by altering where they went. The game made respondents visit new places and select new routes, increasing their encounters with new surroundings and sites. The respondents valued the discovery of new and interesting places. Therefore, the developers of pervasive games could benefit from design decisions that lead players to visit interesting places. This can also have secondary benefits to education and tourism. The focus on physical locations has a drawback, however: some Pokémon GO players complained that it was impossible for them to play because they lived outside large urban centers. This confirms a previous finding that Pokémon GO can reinforce existing geographic advantages and disadvantages [43].

Our study further supported that Pokémon GO requires a low amount of effort to play. For example, the respondents played while doing other things, such as going to work or walking the dog. This made an otherwise routine experience more enjoyable. Based on our data, this was tied to the notion of Pokémon GO being easy to play while doing something else. By implication, for this to be possible, the game should be easy to start and stop and not require a lot of focus from the player, who might be holding onto a leash or groceries while playing. This is also important because playing Pokémon GO while moving from one place to another can be distracting and thus dangerous [32,43].

One central type of behavior change in this study relates to increased physical activity. With our data, conclusions on how long-lasting this behavior change was cannot be made, but it is obvious that at least in the short term, the game increased the physical activity of many respondents. This indicates that pervasive games can be used to promote physical activity. Further, individuals who do not see exercise as enjoyable can use these games to become more active and make physical activities more fun. The game features that motivate players to move are related to two additional aspects: first, Pokémon GO sets concrete goals for players by showing how many Pokémon they need to find and by letting them hatch eggs by walking a specified distance, and second, the game ties the players’ motivation to something they already care about: Pokémon. The other game features may be less important than the connection to a cultural phenomenon that the players already know and love. Previous research has identified goal setting as an important motivator, but the effects of theming have not been explored [8,70].

Finally, we want to highlight the game feature that was strongly tied to all forms of behavior change: game location tied to physical location. Pokémon GO had such a big impact on respondents’ behavior because it is a pervasive game.

### Limitations

Our chosen method for data collection—an online survey—has some general limitations. Sometimes participants respond to open-ended questions in the briefest time possible to minimize what they perceive to be burdensome. To mitigate this, the respondents were asked to recall their incidents and describe them with as much detail as possible before responding. Additionally, following CIT procedures, inquiring about the experience was divided into multiple questions. As such, most of our respondents did give rather detailed responses. However, using an online survey did not allow us to ask follow-up questions regarding, for example, more precise details of the experience such as with whom the respondents played the game. Thus, future research may consider using qualitative interviews.

Another limitation is that the survey was distributed through discussion forums and social media channels; thus, we were likely to reach only players who follow those channels. To mitigate this limitation, we aimed to distribute the survey widely, and it became apparent that the invitation was spread to other channels by the respondents themselves. While our sample was reasonably large for this kind of study, participation biases cannot fully be known due to a lack of knowledge about the population solicited.

What we do know is that the respondents did not represent a global sample, and it is likely that the data is skewed geographically, which might have affected the types of experiences detailed in the answers. Unfortunately, the collected data does not enable a detailed examination of the respondents’ countries. Further, our sample was female dominant (176/262, 67.2%), which is close to the 63% reported by Sonders [73], but other market research indicated a more male dominant gender distribution of Pokémon GO players [74]. Thus, it is likely that women are overrepresented in our sample.

One final limitation of CIT is that it relies on self-reporting; thus, our findings are based on the respondents’ subjective perceptions. The respondents also reported more positive than negative incidents. This is supported by the claim that CIT captures respondents’ outstandingly positive and negative experiences without paying attention to ordinary experiences [60]. This can be due to various reasons, such as individuals’ positive general attitudes toward games, their possible tendency to report positively about games, or the hype and entertainment purpose of Pokémon GO. While we believe that our discovered behavior change types reflect various Pokémon Go experiences, these detailed findings may not be fully applicable in ordinary experiences for this reason.

### Conclusions

The purpose of this study was to examine types of behavior changes pervasive games can induce in their players and kinds of game features that drive different behavior change types. We analyzed the types of behavior changes players of Pokémon GO experienced and which game features are behind the identified behavior changes. The research data was collected from players of the game using CIT [59]. Following a qualitative approach, we analyzed the data by coding and categorizing it.

We contribute to extant knowledge by examining a variety of different behavior change types in one study, while prior studies have focused on a limited set of behavior change types (eg, increased physical activity). Thus, we offer a more comprehensive categorization by identifying different behavior change types enabled by the game and its features. Our second contribution is connecting the behavior changes to game features. We identified 13 game features in Pokémon GO, extending the features found by Meschtscherjakov et al [34] by 4: finding specific Pokémon, using items, Pokémon theme, and game location tied to physical location. Notably, when analyzing the relation between game features and behavior change, we noticed that two features were common to all behavior changes: catching new Pokémon and game location tied to physical location.

## Appendix

Multimedia Appendix 1 Checklist for Reporting Results of Internet E-Surveys (CHERRIES).

Multimedia Appendix 2 Frequency of codes related to behavior change.

## Abbreviations

BCSS: behavior change support system
CHERRIES: Checklist for Reporting Results of Internet E-Surveys
CIT: critical incident technique
IT: information technology

## References

[1] Maddison R, Simons M, Straker L, Witherspoon L, Palmeira A (2013). Active video games: an opportunity for enhanced learning and positive health effects. Cog Technol.

[2] Connolly TM, Boyle EA, MacArthur E, Hainey T, Boyle JM (2012). A systematic literature review of empirical evidence on computer games and serious games. Comput Educ.

[3] Montola M (2005). Exploring the edge of the magic circle: defining pervasive games.

[4] Holt-Lunstad J, Smith TB, Baker M, Harris T, Stephenson D (2015). Loneliness and social isolation as risk factors for mortality: a meta-analytic review. Perspect Psychol Sci.

[5] Matthews CE, George SM, Moore SC, Bowles HR, Blair A, Park Y, Troiano RP, Hollenbeck A, Schatzkin A (2012). Amount of time spent in sedentary behaviors and cause-specific mortality in US adults. Am J Clin Nutr.

[6] Kari T, Piippo J, Frank L, Makkonen M, Moilanen P (2016). To gamify or not to gamify? Gamification in exercise applications and its role in impacting exercise motivation.

[7] Deterding S, Björk S, Nacke L, Dixon D (2013). Designing gamification: creating gameful and playful experiences.

[8] Alahäivälä T, Oinas-Kukkonen H (2016). Understanding persuasion contexts in health gamification: a systematic analysis of gamified health behavior change support systems literature. Int J Med Inform.

[9] Baranowski T, Buday R, Thompson DI, Baranowski J (2008). Playing for real: video games and stories for health-related behavior change. Am J Prev Med.

[10] Edgerton E, Ferdig R (2009). Changing health behavior through games. Handbook of Research on Effective Electronic Gaming in Education.

[11] Klein HK, Myers MD (1999). A set of principles for conducting and evaluating interpretive field studies in information systems. MIS Quarterly.

[12] Venkatesh V, Brown SA, Bala H (2013). Bridging the qualitative-quantitative divide: guidelines for conducting mixed methods research in information systems. MIS Quarterly.

[13] (2019). Niantic Inc.

[14] (2016). Kotaku.

[15] (2016). Newzoo.

[16] (2016). BBC.

[17] Sonders M (2016). SurveyMonkey Intelligence.

[18] Nelson R (2019). SensorTower Inc.

[19] Alha K, Koskinen E, Paavilainen J, Hamari J (2019). Why do people play location-based augmented reality games: a study on Pokémon GO. Comput Human Behav.

[20] Hamari J, Malik A, Koski J, Johri A (2018). Uses and gratifications of Pokémon GO: why do people play mobile location-based augmented reality games?. Int J Human Comput Interact.

[21] Paavilainen J, Korhonen H, Alha A, Stenros J, Koskinen F (2017). The Pokémon GO experience: a location-based augmented reality mobile game goes mainstream.

[22] Kari T, Arjoranta J, Salo M (2017). Behavior change types with Pokémon GO.

[23] (2019). Niantic Inc.

[24] Baranowski T (2016). Pokémon Go, go, go, gone?. Games Health J.

[25] Kari T, Makkonen M (2014). Explaining the usage intentions of exergames. http://urn.fi/URN:NBN:fi:jyu-201501151118.

[26] (2019). Niantic Inc.

[27] de Souza e Silva A (2016). Pokemon Go as an HRG: mobility, sociability, and surveillance in hybrid spaces. Mob Media Commun.

[28] Hjorth L (2017). Pokémon GO: playful phoneurs and the politics of digital wayfarers. Mob Media Commun.

[29] Baranowski T, Lyons EJ (2019). Scoping review of Pokémon Go: comprehensive assessment of augmented reality for physical activity change. Games Health J.

[30] Althoff T, White RW, Horvitz E (2016). Influence of Pokémon Go on physical activity: study and implications. J Med Internet Res.

[31] Broom DR, Flint SW (2018). Gotta catch 'em all: impact of Pokémon GO on physical activity, sitting time, and perceptions of physical activity and health at baseline and three-month follow-up. Games Health J.

[32] Chong Y, Sethi DK, Loh CHY, Lateef F (2018). Going forward with Pokemon Go. J Emerg Trauma Shock.

[33] Howe KB, Suharlim C, Ueda P, Howe D, Kawachi I, Rimm EB (2016). Gotta catch'em all! Pokémon GO and physical activity among young adults: difference in differences study. BMJ.

[34] Meschtscherjakov A, Trösterer S, Lupp A, Tscheligi M (2017). Pokémon WALK: persuasive effects of Pokémon GO game-design elements.

[35] Nigg CR, Mateo DJ, An J (2017). Pokémon GO may increase physical activity and decrease sedentary behaviors. Am J Public Health.

[36] LeBlanc AG, Chaput J (2016). Pokémon Go: a game changer for the physical inactivity crisis?. Prev Med.

[37] Wong FY (2017). Influence of Pokémon Go on physical activity levels of university players: a cross-sectional study. Int J Health Geogr.

[38] Tateno M, Skokauskas N, Kato TA, Teo AR, Guerrero APS (2016). New game software (Pokémon Go) may help youth with severe social withdrawal, hikikomori. Psychiatry Res.

[39] Nikou S, Tarvoll J, Öörni A (2018). Impact of playing Pokémon go on wellness.

[40] McCartney M (2016). Game on for Pokémon Go. BMJ.

[41] Joseph B, Armstrong DG (2016). Potential perils of peri-Pokémon perambulation: the dark reality of augmented reality?. Oxf Med Case Reports.

[42] Serino M, Cordrey K, McLaughlin L, Milanaik RL (2016). Pokémon Go and augmented virtual reality games: a cautionary commentary for parents and pediatricians. Curr Opin Pediatr.

[43] Colley J, Thebault-Spieker A, Lin D, Degraen B (2017). The geography of Pokémon GO: beneficial and problematic effects on places and movement.

[44] Yang C, Liu D (2017). Motives matter: motives for playing Pokémon Go and implications for well-being. Cyberpsychol Behav Soc Netw.

[45] Kari T (2016). Pokémon GO 2016: exploring situational contexts of critical incidents in augmented reality. J Virtual Worlds Res.

[46] Zach F, Tussyadiah P, Schegg R, Stangl B (2017). To catch them all? The (un) intended consequences of Pokémon GO on mobility, consumption, and wellbeing. Information and Communication Technologies in Tourism.

[47] Oinas-Kukkonen H (2012). A foundation for the study of behavior change support systems. Pers Ubiquit Comput.

[48] Fogg BJ (2003). Persuasive Technology Using Computers to Change What We Think and Do.

[49] Connolly TM, Boyle EA, MacArthur E, Hainey T, Boyle JM (2012). A systematic literature review of empirical evidence on computer games and serious games. Comput Educ.

[50] Michie S, Johnston M (2012). Theories and techniques of behaviour change: developing a cumulative science of behaviour change. Health Psychol Rev.

[51] Michie S, Richardson M, Johnston M, Abraham C, Francis J, Hardeman W, Eccles MP, Cane J, Wood CE (2013). The behavior change technique taxonomy (v1) of 93 hierarchically clustered techniques: building an international consensus for the reporting of behavior change interventions. Ann Behav Med.

[52] Davis R, Campbell R, Hildon Z, Hobbs L, Michie S (2015). Theories of behaviour and behaviour change across the social and behavioural sciences: a scoping review. Health Psychol Rev.

[53] Stenros J, Montola M, Mäyrä F (2007). Pervasive games in Ludic society.

[54] (2004). 42 Entertainment.

[55] (2004). Bungie.

[56] (2001). It's Alive Mobile Games AB!.

[57] Stenros J, Montola M, Waern A, Jonsson S (2007). Momentum Evaluation Report.

[58] Klein HK, Myers MD (1999). A set of principles for conducting and evaluating interpretive field studies in information systems. MIS Quarterly.

[59] Flanagan JC (1954). The critical incident technique. Psychol Bull.

[60] Gremler DD (2016). The critical incident technique in service research. J Serv Res.

[61] Edvardsson B, Roos I (2001). Critical incident techniques. Int J Serv Ind Mgmt.

[62] Gogan J, McLaughlin M, Thomas D (2014). Critical incident technique in the basket. https://aisel.aisnet.org/icis2014/proceedings/ResearchMethods/2/.

[63] Grove SJ, Fisk RP (1997). The impact of other customers on service experiences: a critical incident examination of “getting along”. J Retailing.

[64] Meuter ML, Ostrom AL, Roundtree RI, Bitner MJ (2000). Self-service technologies: understanding customer satisfaction with technology-based service encounters. J Marketing.

[65] Bitner MJ, Booms BH, Tetreault MS (2018). The service encounter: diagnosing favorable and unfavorable incidents. J Marketing.

[66] Eysenbach G (2004). Improving the quality of Web surveys: the Checklist for Reporting Results of Internet E-Surveys (CHERRIES). J Med Internet Res.

[67] Guest G, MacQueen K (2012). Applied Thematic Analysis.

[68] Charmaz K (2014). Constructing Grounded Theory. 2nd Edition.

[69] Oduor M, Alahäivälä T, Oinas-Kukkonen H (2014). Persuasive software design patterns for social influence. Pers Ubiquit Comput.

[70] Edwards EA, Lumsden J, Rivas C, Steed L, Edwards LA, Thiyagarajan A, Sohanpal R, Caton H, Griffiths CJ, Munafò MR, Taylor S, Walton RT (2016). Gamification for health promotion: systematic review of behaviour change techniques in smartphone apps. BMJ Open.

[71] Williams D (2003). The video game lightning rod. Inform Commun Soc.

[72] Humphreys L (2016). Involvement shield or social catalyst: Thoughts on sociospatial practice of Pokémon GO. Mob Media Commun.

[73] Sonders M (2016). SurveyMonkey Intelligence.

[74] Iqbal N (2020). Business of Apps.

