# Supplementary material for: Exploring Features of the Pervasive Game Pokémon GO That Enable Behavior Change: Qualitative Study
Source: JMIR Serious Games. 2020 May 25;8(2):e15967. doi: 10.2196/15967 (PMC7281148; doi:10.2196/15967)
Supplement: Multimedia Appendix 1 [file games_v8i2e15967_app1.pdf]

Item Category	Checklist Item	Explanation
**Design**		
	Describe survey design	The survey's target population was players of *Pokémon GO*, meaning that the survey used a convenience sample. The survey description mentioned it was "meant for people of all age who play or have played *Pokémon Go*."
**IRB approval and informed consent process**		
	IRB approval	This study was not submitted for IRB review.
	Informed consent	The participants were informed of the length of the survey, the number of questions, the purpose of the study, and the university organizing the data collection.
	Data protection	The survey did not include identifiable information, and all participants were anonymous.<br><br>The database containing the responses was protected with username and password login.
**Development and pre-testing**		
	Development and testing	The survey was developed using the LimeSurvey 2.05+ software.<br><br>The questionnaire was pre-tested with 10 early adopters of *Pokémon GO.* This included both usability and technical functionality testing.
**Recruitment process and description of the sample**		

**having access to the questionnaire**		
	Open survey versus closed survey	The survey was open to all visitors.
	Contact mode	Initial contact with potential participants was made on the internet.
	Advertising the survey	The questionnaire was distributed via different online forums (eg, related to gaming, *Pokémon GO*, lifestyle, wellness, culture, cooking, sports, cuisine) and social networks (Facebook, Twitter, and Reddit).<br><br>The wording of the announcement was as follows:<br><br>[Title] A survey on *Pokémon GO* playing experiences<br><br>We are conducting a survey on the playing experiences people have with *Pokémon GO*.<br>The survey is meant for people of all age who have played *Pokémon GO*.<br>It takes about 10 minutes to answer the survey.<br>Answering is totally anonymous, and the responses will be dealt in strictest confidence and with principles for good scientific and ethical conduct.<br>Please spare a few minutes and answer.<br>A warm thank you for your interest in our survey.<br>The survey can be accessed via this link: [link].
**Survey administration**		

	Web/Email	The survey was administrated through the LimeSurvey 2.05+ software. The responses were automatically saved into a database.
	Context	The survey was located on its own website. The website had no other content besides the survey.
	Mandatory/voluntary	Participation in the survey was voluntary.
	Incentives	No incentives for participation were offered.
	Time/Date	The survey was open from 2016-07-16 to 2016-09-15.
	Randomization of items or questionnaires	There were no such question items where randomization was needed.
	Adaptive questioning	Questions related to critical incidents were only asked from the respondents who had experience playing *Pokémon GO*.
	Number of items	The survey had 26 questions: 6 demographic and 20 related to the *Pokémon GO* experience. Some questions were presented for the purpose of another study.<br><br>The number of items per page varied from 2 to 12.
	Number of screens (pages)	The survey had 4 pages.
	Completeness check	A completeness check was carried out after the survey was closed. Only complete responses (responses from participants who had answered the whole survey) were used.<br><br>An “I cannot say” option was provided where applicable to avoid forced responses.

	Review step	The respondents were able to change their answers using a Back button. No summary or review of the responses was shown at the end of the survey.
**Response rates**		
	Unique site visitors	Unique site/survey visitors were not tracked.<br><br>IP address tracking and setting cookies were not used.
	View rate (ratio of unique survey visitors to unique site visitors)	Unique site/survey visitors were not tracked.
	Participation rate (ratio of unique visitors who agreed to participate / unique first survey page visitors)	The participation rate was 62.3%. Of the 884 first page visits, 551 led to starting the survey.<br><br>Note: as we did not track unique visitors, a respondent could have visited the first (or another) page more than once.
	Completion rate (ratio of users who finished the survey to users who agreed to participate)	The completion rate was 49%. In total, we received 270 complete responses and 281 incomplete responses.<br><br>Note: as we did not track unique visitors, a respondent could have started the survey and abandoned it, but then returned later to complete it.
**Preventing multiple entries from the same individual**		
	Cookies used	No cookies were used.
	IP check	No IP tracking was used.
	Log file analysis	No log file analysis was used for identification of multiple entries.

		We consider the risk of a single respondent answering multiple times very low.
	Registration	The survey was open to all visitors.
**Analysis**	Handling of incomplete questionnaires	Only completed questionnaires were analyzed.
	Questionnaires submitted with an atypical timestamp	N/A
	Statistical correction	The survey did not aim for statistical representation of a specific population.
