# Supplementary material for: Exploring Features of the Pervasive Game Pokémon GO That Enable Behavior Change: Qualitative Study
Source: JMIR Serious Games. 2020 May 25;8(2):e15967. doi: 10.2196/15967 (PMC7281148; doi:10.2196/15967)
Supplement: Multimedia Appendix 2 [file games_v8i2e15967_app2.pdf]

Multimedia Appendix 2: Frequency of codes related to behavior change.

	n
**Added activity in life**	
Break from routine	12
Going out	34
Playing alone	13
Playing as a reward	1
**Enhancing routines**	
Walking the dog	2
Playing while doing something else	31
**Exploration**	
Exploration	13
Just moved here	2
Use of public spaces	3
**Increased physical activity**	
Exercise	90
Exergames	1
**Strengthening social bonds**	
Playing for somebody else	1
Reason for playing: Social pressure	8
Spending time with family	45
Spending time with friends	63
Spending time with significant other	26
**Lowering social barriers**	
Community	23
Community: online	6
Competition or player versus player gameplay	14
Groups of people	36
Making new friends	14
Meeting neighbors	4
Meeting new people	64
Meetup	3
Negative social experience	7
People of different ages playing or meeting	17
Socialization	73
Teaching the game to others	9
Teams	15
Teamwork	12
**Increased positive emotional expression**	
Helping others	8
Positive change in other people	12
Shared excitement/interest	30
**Self-treatment**	
Helps with anxiety or depression	5

Improved mood	10
Treatment	8
